# Proton Conductivity Enhancement at High Temperature on Polybenzimidazole Membrane Electrolyte with Acid-Functionalized Graphene Oxide Fillers

**DOI:** 10.3390/membranes12030344

**Published:** 2022-03-19

**Authors:** Raja Rafidah Raja Sulaiman, Rashmi Walvekar, Wai Yin Wong, Mohammad Khalid, Ming Meng Pang

**Affiliations:** 1 Faculty of Innovation and Technology, School of Computer Science and Engineering, Taylor’s University, Subang Jaya 47500, Selangor, Malaysia; rafeedahrosset@gmail.com (R.R.R.S.); mingmeng.pang@taylors.edu.my (M.M.P.); 2 Department of Chemical Engineering, School of New Energy and Chemical Engineering, Xiamen University Malaysia Campus, Jalan Sunsuria, Bandar Sunsuria, Sepang 43900, Selangor, Malaysia; 3 Fuel Cell Institute, Universiti Kebangsaan Malaysia, UKM, Bangi 43600, Selangor, Malaysia; 4 Graphene & Advanced 2D Materials Research Group (GAMRG), School of Engineering and Technology, Sunway University, No. 5, Jalan Universiti, Bandar Sunway, Petaling Jaya 47500, Selangor, Malaysia

**Keywords:** energy conversion, fuel cell, proton exchange membrane, polybenzimidazole, graphene oxide

## Abstract

Graphene oxide (GO) and its acid-functionalized form are known to be effective in enhancing the proton transport properties of phosphoric-acid doped polybenzimidazole (PA-doped PBI) membranes utilized in high-temperature proton exchange membrane fuel cells (HTPEMFC) owing to the presence of proton-conducting functional groups. This work aims to provide a comparison between the different effects of GO with the sulfonated GO (SGO) and phosphonated GO (PGO) on the properties of PA-doped PBI, with emphasis given on proton conductivity to understand which functional groups are suitable for proton transfer under high temperature and anhydrous conditions. Each filler was synthesized following existing methods and introduced into PBI at loadings of 0.25, 0.5, and 1 wt.%. Characterizations were carried out on the overall thermal stability, acid doping level (ADL), dimensional swelling, and proton conductivity. SGO and PGO-containing PBI exhibit better conductivity than those with GO at 180 °C under anhydrous conditions, despite a slight reduction in ADL. PBI with 0.5 wt.% SGO exhibits the highest conductivity at 23.8 mS/cm, followed by PBI with 0.5 wt.% PGO at 19.6 mS/cm. However, the membrane with PGO required a smaller activation energy for proton conduction, thus less energy was needed to initiate fast proton transfer. Additionally, the PGO-containing membrane also displayed an advantage in its thermal stability aspect. Therefore, considering these properties, it is shown that PGO is a potential filler for improving PBI properties for HTPEMFC applications.

## 1. Introduction

The proton exchange membrane fuel cell (PEMFC) holds a promising potential for clean energy conversion through the utilization of hydrogen fuel to produce electricity with near-zero production of harmful pollutants. Its energy input to electrical output conversion efficiency could reach around 40 to 45%, which is comparable to that of a gasoline/battery hybrid. The operating temperature of the PEMFC varies from 60 to 180 °C [1]. High-temperature proton exchange membrane fuel cells (HTPEMFC) are PEMFCs that operate beyond the boiling temperature of water (>100 °C), which have several advantages including improved catalyst tolerance towards carbon monoxide (CO) poisoning and simplified hydration management. Components in HTPEMFC differ from the lower temperature PEMFC as they employ a polybenzimidazole (PBI) membrane as its proton exchange membrane (PEM) in the membrane electrode assemble (MEA), rather than the Nafion membrane. Additionally, phosphoric acid (PA) functions as the proton carrier in HTPEMFC due to it being a low volatile acid with effective proton conducting ability at higher temperatures [2,3]. Typically, the phosphoric acid is embedded in the PBI membrane as PA-doped PBI to be utilized as the PEM in the HTPEMFC.

In terms of its physicochemical properties that make the PBI attractive as a PEM for HTPEMFC, the membrane is a thermally, mechanically, and chemically stable polymer. Several derivatives of PBI with various functional groups have been synthesized. The poly[2,2-(m-phenylene)-5,5-bibenzimidazole] is a common PBI derivative without any branch functional groups [4]. Two N-H sites on the imidazole rings of each PBI repeat unit can bind to two phosphoric acid molecules, resulting in the acid doping level (ADL) of 2 mol PA per repeat unit of PBI (mol PA/rpu PBI). Additional acid molecules occupy the free spaces between the PBI polymer chains. As more PA is bound and contained within the PBI matrix, the ADL increases. Higher ADL leads to better proton conductivity of the PA-doped PBI membrane. In addition, the proton conductivity is also enhanced with temperature. Thus, the higher ADL in PBI offers fast proton transfer at the HTPEMFC operating temperature between 110 to 180 °C [3,5,6]. However, the downside of high ADL is the very swollen membrane with weakened mechanical properties, which could pose a high risk of rupturing during HTPEMFC operation. Researchers have been attempting to achieve the appropriate ADL content in the PBI that gives sufficiently high proton conductivity while maintaining its physical strength for practical applications in HTPEMFC. Among the methods to improve the PEM properties of PBI are the addition of organic or inorganic fillers into the PBI membrane [3].

**Figure 1:**
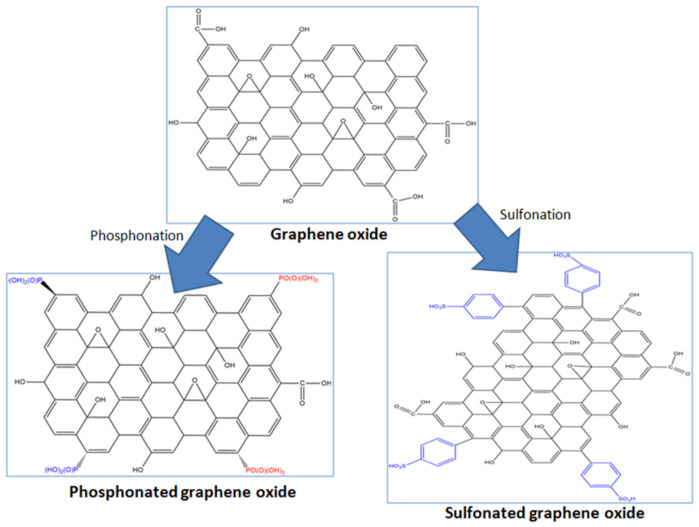
Schematic diagram of graphene oxide and its sulfonated and phosphonated forms.

Graphene oxide (GO) are graphene sheets containing oxygen-containing functional groups such as alkoxy, epoxides, hydroxides, and carboxylic acids that can participate in proton transfer. The GO maintains the large surface area of graphene and is electrically insulating [7]. GO can also be functionalized further with acidic functional groups such as sulfonic or phosphonic acid, which are much stronger proton-conducting functional groups. A schematic representation of the GO and its acid-functionalized derivative is presented in Figure 1. In recent years, GO and its acid-functionalized derivatives have been investigated in PBI-based membranes for HTPEMFC applications. The addition of GO-based fillers into PBI may increase ADL slightly, but in some cases may also result in no change to the ADL. However, significant improvements to the proton conductivity were observed. For instance, Uregen et al. [8] determined a 79% enhancement to the proton conductivity of PBI with GO at 180 °C under anhydrous conditions, despite no change to the acid uptake of the membrane. Besides GO, sulfonated graphene oxide (SGO) has also been extensively studied as a filler for PBI. Cai et al. [9] found that SGO was better at enhancing the conductivity of PBI compared to GO. A recent study by Yusoff et al. [10] also showed that the conductivity of PA-doped PBI with SGO was better than pure PBI, attributed to the sulfonic acid groups participating in the proton transfer in the membrane. A new study by Abouzari-Loft et al. [11] introduced phosphonated graphene oxide (PGO) into pyridine-functionalized PBI. PGO not only facilitates proton transfer in Py-PBI compared to pure Py-PBI, but also helps maintain cell performance and conductive stability for over 20 h. Aside from conductivity enhancement, these GO-based fillers also contribute to the dimensional and mechanical stability of the PA-doped PBI. These studies have shown the benefit offered by GO, SGO, and PGO towards PBI, and the performance of these modified membranes could be better than pure PBI by giving the membrane appropriate levels of ADL and sufficiently high proton conductivity. However, no direct comparison in the properties has been made between PBI containing PGO and those with GO as well as with SGO. Phosphonic groups in PGO have amphoteric characteristics and can form strong hydrogen bonds, which may offer better proton transferability under anhydrous conditions [12,13]. Whereas SGO contains a strong proton conductive sulfonic acid, the conducting ability of sulfonic acid is more dependent on water content and may poorly ionize in anhydrous conditions [14,15]. Direct comparison on the effects of PGO with those of SGO towards PA-doped PBI under similar operating conditions will provide insight on which functional group is potentially more suitable for facilitating proton transfer under the high temperature and anhydrous conditions of HTPEMFC.

Therefore, to understand the effects of the different functional groups on GO towards properties of PA-doped PBI, this work aims to conduct a comparison between the PEM properties of the PA-doped PBI membranes modified with GO, SGO, or PGO fillers. GO, SGO and PGO were synthesized following the procedures as previously described in the literature, which was then embedded into the PBI as fillers in the membrane. Focus is given on the proton conductive effects of GO, SGO, and PGO on similar PBI membranes, while other physicochemical properties such as ADL, dimensional swelling, and thermal properties are also investigated. As observed from the results, ADL appeared to reduce upon addition of either GO, SGO or PGO into PBI; however, the fillers contribute to an increase in proton conductivity. SGO offers the largest proton conductivity increment in the PA-doped PBI, but also required the highest activation energy. PGO-containing PBI membranes have slightly lower conductivity than the SGO-containing membranes, yet has a smaller requirement for activation energy under anhydrous conditions, thus showing the advantage of PGO towards fast proton conduction. 

## 2. Materials and Methods

### 2.1. Materials

Polybenzimidazole (PBI) powder (viscosity at 6000 Pa∙s in 10 wt.% DMAc, standard temperature, T_g_ = ~400 °C) was purchased from Shanghai Shengjun Plastic Technology. Graphite flakes (99% carbon basis, −325 mesh particle size, ≥99%, natural), N,N-dimethylacetamide (DMAc), sulfuric acid (H_2_SO_4_) (98%), potassium permanganate (KMnO_4_), sodium nitrate (NaNO_3_), sodium nitrite (NaNO_2_), hydrogen peroxide (H_2_O_2_), sulfanilic acid (SA), 37% hydrochloric acid (HCl), sodium hydroxide (NaOH) pellets, polyphosphoric acid (PPA) and 85% orthophosphoric acid (PA) were purchased from Sigma Aldrich (St. Louis, MO, USA).

### 2.2. Synthesis of GO

GO was synthesized from graphite flakes following the Hummers method [16]. Graphite flakes were first pre-oxidized by mixing with 12 mL of 98% H_2_SO_4_ at 80 °C for 4 h, which was then cooled and sonicated for 5 h at room temperature. Pre-oxidized graphite solid was obtained after dilution and filtration. The solids were placed in 120 mL of 98% H_2_SO_4_, followed by slow addition of KmnO_4_ under stirring at room temperature for 2 h. The mixture was then diluted and mixed with 20 mL of 30% H_2_O_2_ to terminate the oxidation reaction. The solution was separated into 15 mL small batches to be centrifuged and washed with dilute HCl (ratio of HCl: H_2_O at 1:9) and deionized (DI) water repeatedly until a light-yellow supernatant was obtained. The concentrated GO solution was diluted with DI water to a concentration of 1 mg/mL GO.

### 2.3. Synthesis of SGO and PGO

GO is functionalized to SGO using sulfanilic acid-based aryl diazonium salt based on the procedures by Li et al. [17]. Briefly, 100 mg of sulfanilic acid (SA) and 40 mg of sodium nitrite (NaNO_2_) were added into a solution of HCl (ratio HCl: H_2_O at 1:5), followed by the addition of 1 mL of 2 wt.% sodium hydroxide (NaOH) solution. The mixture was kept in an ice bath at temperatures of approximately 10 °C under stirring at 300 rpm for 15 min, which would lead to the formation of arenediazonium salts. The salts were then mixed with 100 mL of GO solution (concentration 1 mg/mL GO) for 4 h in the ice bath. The solution was centrifuged in small batches and repeatedly washed with DI water until the pH of the supernatant was close to neutral.

Functionalization of GO to PGO is according to the procedures by Abouzari-Lotf [11]. Phosphoric acid (PA) and polyphosphoric acid (PPA) were mixed at a 5:1 (PA:PPA) ratio through sonication. the pH of the acid mixture was adjusted to pH 5 before mixing with 150 mL of 1 mg/mL of GO solution. The mixture was heated to around 95 °C and the reaction was carried out for 8 h. The solution in small batches was centrifuged under 2 °C to allow the solid to separate from the supernatant. Centrifugation was conducted repeatedly for at least three times. Dried GO, SGO, and PGO sheets/flakes were obtained by drying the solution in a gravity convection oven under 85 °C.

### 2.4. PBI and Composite PBI Membrane Preparation

PBI solution was prepared by dissolving 2 wt.% PBI powder in DMAc in a Teflon-lined autoclave at 190 °C for 24 h without stirring. GO, SGO, and PGO fillers were added into the PBI solutions at a concentration of 0.25, 0.5, and 1 wt.% of the weight of PBI powder. Fillers were dispersed through ultrasonication in an ultrasonic water bath until full dispersion in the PBI solution. The solutions were then cast onto clean glass Petri dishes, which were placed in a gravity convection oven at 65 °C for 48 h to evaporate the solvents and allow the membrane to form slowly. The obtained membrane film was carefully peeled off the petri dish with the aid of DI water. The membranes were boiled in DI water for 2 h, followed by drying under 165 °C for 14 h to remove trace solvents [11]. Samples are denoted as PBI-*x-y*, where *x* is the filler type (GO, SGO, and PGO) and *y* is the filler concentration (0.25, 0.5, 1) in wt.%. A PBI membrane without filler was also prepared as a control.

### 2.5. Physicochemical Characterization

Chemical characterization and bonding in fillers and membranes were analyzed using attenuated total reflection Fourier transform infrared spectroscopy (ATR-FTIR) (Perkin Elmer, Spectrum Two, Waltham, MA, USA) within the wavelength range of 450–4000 cm^−1^. Membrane morphology was observed through field emission scanning electron microscopy (FESEM) (FEI Quanta 400F, FEI company, Hillsboro, OR, USA) under room temperature with built-in energy dispersive X-ray spectroscopy (EDX). Films were sputter-coated with platinum using Quorum Q150R S Plus sputter coater to prevent high charging and enable clear focus under high magnification. The crystallinity and amorphous aspects of membranes at the middle filler loading level were determined using X-ray diffraction (XRD) using a D8 Advance XRD (Bruker AXS, Billerica, MA, USA) with 2ϴ = 5–80° at room temperature. Thermal properties of all un-doped membranes were analyzed by using thermogravimetric analysis (TGA) (PerkinElmer, TGA 8000, Waltham, MA, USA) under nitrogen from 30 to 800 °C with a heating rate of 10 °C /min. TGA is considered a common method for analyzing the overall thermal stability of PEM membranes.

### 2.6. PA Doping, ADL, Dimensional Swelling, and Acid Leaching Test

Acid doping of PBI membranes was carried out at room temperature. Membranes were first dried in a vacuum oven at 85 °C to eliminate absorbed water for 4 h when the weight of the dry membranes became constant. The membranes were then immersed in 85% orthophosphoric acid for 48 h. The doped membranes were carefully wiped with filter paper to remove excess surface acid, followed by drying in a vacuum oven at 75 °C for 4 h to remove excess moisture. The ADL of the doped membranes is calculated using the following Equation [10]:

(1) $$\mathrm{Acid}\;\mathrm{doping}\;\mathrm{level}:\;ADL\;\left(\frac{mol\;PA}{rpu\;PBI}\right)=\frac{W_{doped}-W_{dry}}{W_{dry}}\times \left(\frac{MW\;PBI}{MW\;\mathrm{PA}}\right)$$

where *W_doped_* is the mass of the doped membrane and *W_dry_* is the mass of dry membrane in milligrams (mg). The molecular weights of the PBI repeat unit (MW PBI) and PA molecule (MW PA) are 308 and 98 g/mol, respectively. Further, the percentage swelling ratio (%SR) in terms of the change in membrane’s length and thickness was also calculated through Equations (2) and (3):

(2) $$\mathrm{Length}\;\mathrm{swelling}:\;\%SR_{L}=\frac{L_{doped}-L_{dry}}{L_{dry}}\times 100\%$$

(3) $$\mathrm{Thickness}\;\mathrm{swelling}:\;\%SR_{t}=\frac{t_{doped}-t_{dry}}{t_{dry}}\times 100\%$$

where *L_doped_*, and *t_doped_* are the PA-doped lengths and thickness of the membrane, respectively, while *L_dry_* and *t_dry_* are the length and thickness of dry membranes. All dimensional measurements are in centimeters (cm). The thickness of the dry membranes is kept at approximately 81 ± 7 μm.

The acid leaching test was conducted based on the procedures in [18,19] for selected membranes that showed the highest proton conductivity. The membranes were hung above boiling water for a total duration of 5 h. The change in weight of the membranes was measured in an interval of 30 min in the first 3 h, and an hour interval for the subsequent hour. PA loss was calculated using the following Equation:

(4) $$\mathrm{Loss}\;\mathrm{in}\;\mathrm{PA}:\;\%Loss=\frac{W_{initial}-W_{leached}}{W_{doped}}\times 100\%$$

where *W_initial_* is the initial weight of the doped membrane before testing, *W_leached_* is the weight of the leached membrane, and *W_doped_* is the calculated doped weight of the membrane determined from ADL measurements. ADL, swelling, and leaching experiments were repeated three times to obtain the average and measure the result’s reproducibility.

### 2.7. Proton Conductivity Measurements

Electrochemical impedance spectroscopy (EIS) (Autolab PGSTAT128N frequency response analyzer) was conducted to investigate the electrochemical properties and determine the proton conductivity of the acid-doped PBI membranes. Samples measuring approximately 1.6 cm in diameter were sandwiched in between two stainless steel electrodes within a measurement cell through an alternative current mode. Frequency ranged from 0.1 Hz to 1000 kHz with a signal amplitude of 0.1 V. Measurements were conducted at a temperature range from 110 to 180 °C under anhydrous conditions. The Nyquist plot was generated for each measurement and was analyzed using the NOVA software (NOVA 1.11). The membrane resistance (*R_s_*) was determined through electrochemical circle fitting of impedance data in Nyquist plots, which was used to calculate the proton conductivity using Equation (5):

(5) $$\sigma \left(\frac{S}{cm}\right)=\frac{t}{R_{s}A}$$

where *σ* is the proton conductivity in Siemens per centimeter (S/cm), *t* is the sample thickness (cm), *A* is the surface area (cm^2^), and *R_s_* is the electrolyte resistance (Ω). The experiment is repeated three times to average the results and measure the reproducibility.

## 3. Results

### 3.1. FTIR

**Figure 2:**
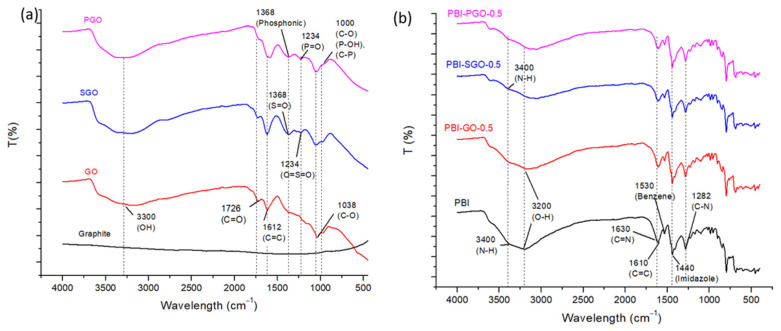
Fourier transform infrared (FTIR) spectra of (**a**) fillers and (**b**) membranes.

FTIR spectra of the fillers and composite membranes containing 0.5 wt.% of fillers are shown in Figure 2a,b, respectively. Graphite spectrum shows the absence of oxygen-containing functional groups [20]. GO displays the stretching vibrations of epoxide groups, C–O (1038 cm^−1^), C=C bonds (1612 cm^−1^), and the C=O of the carboxylic acid or carbonyl moiety (1726 cm^−1^). The broad peak between 2200 to 3500 cm^−1^ is attributed to the stretching vibration of hydroxyl (–OH) groups on the GO plane and hydrogen-bonded –OH of water [16,21,22]. Upon sulfonation to SGO, new medium peaks at 1234 and 1368 cm^−1^ represent the symmetric vibration of the O=S=O and the S=O bond from sulfonic acid groups. The peak at 1612 cm^−1^ is also sharper in the SGO spectrum, which shows the presence of additional C=C bonds due to the attached phenyl rings derived from the aryl diazonium salt used for sulfonation [23,24,25,26]. In the PGO spectrum, the slight shift towards a higher wavelength at 1038 cm^−1^ may be attributed to the overlapping of the P–O, P–OH, and C–P characteristic peaks with the epoxide peak. The small peak at 1234 cm^−1^ for PGO may be attributed to the P=O group. The peak at 1368 cm^−1^ may be due to the existence of phosphonic or phosphoric groups on PGO. Additionally, the intensity of the carboxylic acid group in PGO is lower, as the more ionizable phosphonic acid replaces the carboxylic acids at the plane edges [27,28,29,30]. These results confirm the sulfonation and phosphonation of GO have occurred. Furthermore, the –OH band is broader for SGO and PGO compared to GO, which is due to the more hydrophilic property of the fillers.

FTIR spectra of the un-doped PBI membranes are as shown in Figure 2b. Pure PBI membrane displays characteristic peaks for the stretching vibrations of C–N (1282 cm^−1^), C=C (1610 cm^−1^), and C=N (1630 cm^−1^). The peak at 1440 cm^−1^ is the in-plane deformation of the imidazole rings. The N–H functional groups are indicated in the broad peak at 3400 cm^−1^ [31,32,33]. These groups are crucial for the absorption of phosphoric acid. Additionally, PBI is also a hydrophilic polymer that is sensitive to moisture [34,35]. Thus, the O–H broad peak at 3200 cm^−1^ is present as the absorbed moisture. The introduction of GO caused a slight reduction in the intensity of the N−H broad peak (3400 cm^−1^). The membrane with SGO and PGO also displayed smaller intensity on the same peak. Furthermore, the intensity of C=N was also reduced for the composite PBIs. This could indicate that hydrogen bonding formed between the oxygen-containing functional groups from GO, SGO, and PGO with N–H. In SGO and PGO, acid–base interactions would form between the sulfonic acid or phosphonic acid groups with the N–H [11,36,37]. Moisture is still present in the composite PBI to some extent, as shown from the broad peak at 3200 cm^−1^ since the hydrophilic GO, SGO, and PGO could also absorb water molecules. However, the intensity of the peak between 3200 and 3400 cm^−1^ is affected by the hydrogen bonding and acid-base interactions between the PBI polymer and the fillers. Overall, changes to the PBI spectrum confirm that GO, SGO, and PGO have been embedded in the PBI.

### 3.2. FESEM and EDX Mapping

**Figure 3:**
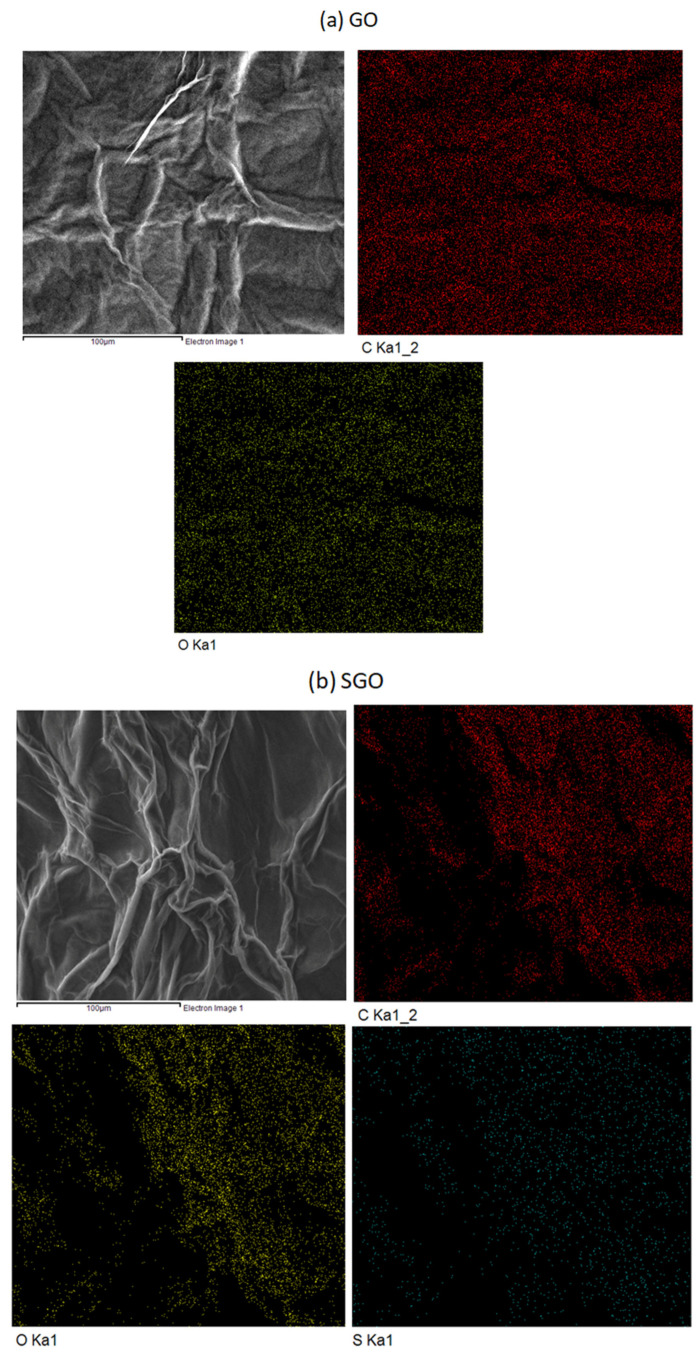

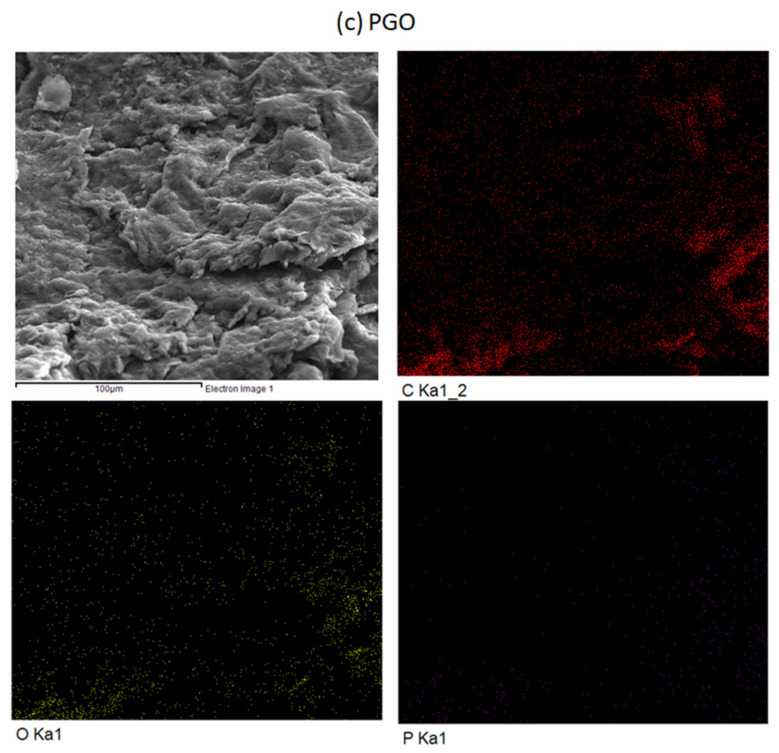
Field emission scanning electron microscopy (FESEM)images and energy dispersive X-ray spectroscopy (EDX) mapping of fillers (**a**) graphene oxide (GO), (**b**) sulfonated graphene oxide (SGO), and (**c**) phosphonated graphene oxide (PGO).

The surface morphology of GO is as shown in Figure 3a, displaying a wavy, sheet-like surface, confirming that the GO has a two-dimensional sheet structure with a high surface area. The appearance of the GO is consistent with those prepared through the Hummers method by Rattana et al. [21] and Zelechowska et al. [28]. The surface morphologies of SGO and PGO are shown in Figure 3b,c, respectively. SGO and PGO show more wrinkles and stacking of the sheets, due to the interactions via hydrogen bonding between the oxygen-containing functional groups, sulfonic acid, and phosphonic acid on the surface of SGO and PGO, making it more disordered [23,29,30]. Energy dispersive X-ray spectrometry (EDX) mapping shows the distribution of the elements on the surface of GO, SGO, and PGO. Carbon is prominent on all the fillers, while oxygen elements appear to present in smaller amounts in PGO indicating the lower quantity of oxygen-containing functional groups in PGO. The surface of SGO also shows the presence of sulfur elements derived from sulfonic acid. Phosphoric groups appear in small quantities over the PGO, where the phosphonic acid groups are present. Nevertheless, the acid-functionalized fillers retain the graphene-like structure of GO, and the changes to the morphology as well the presence of additional sulfur and phosphorus confirm that sulfonation and phosphonation have occurred.

**Figure 4:**
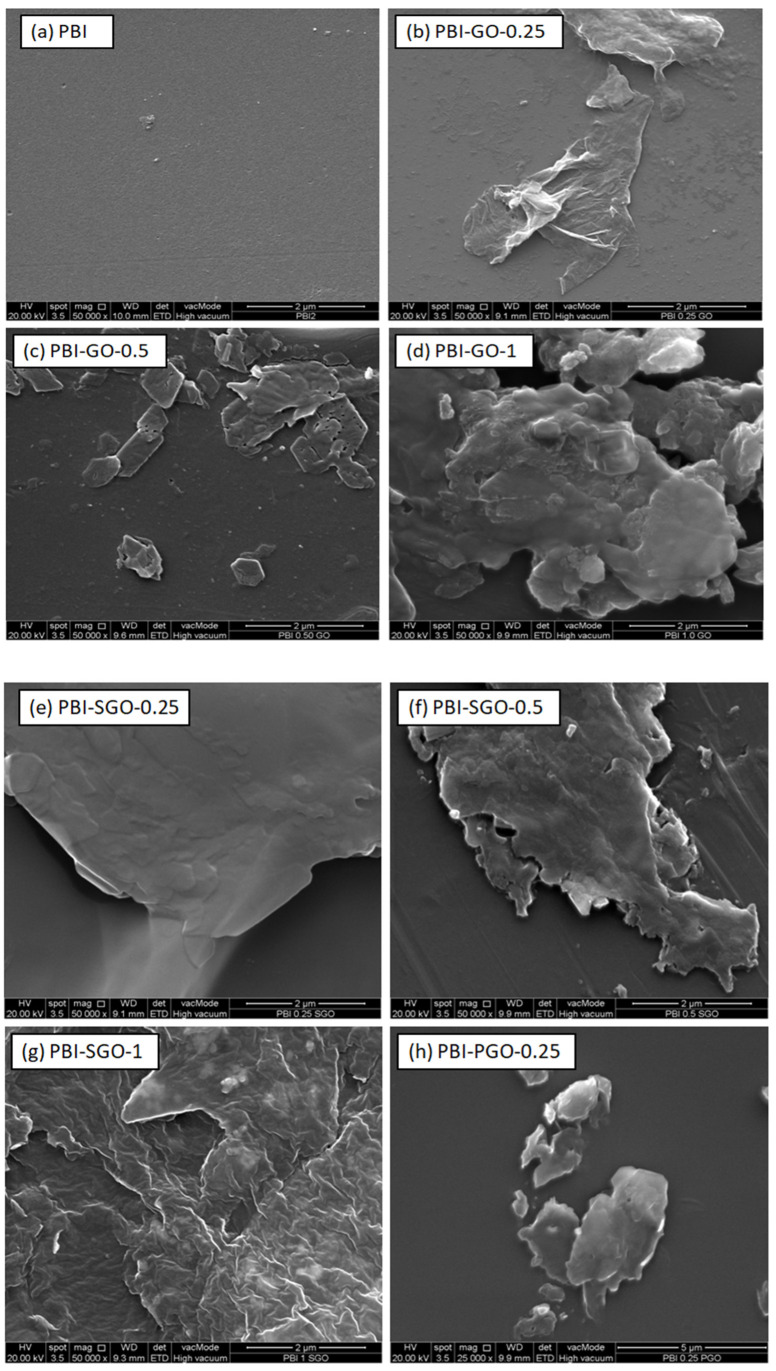

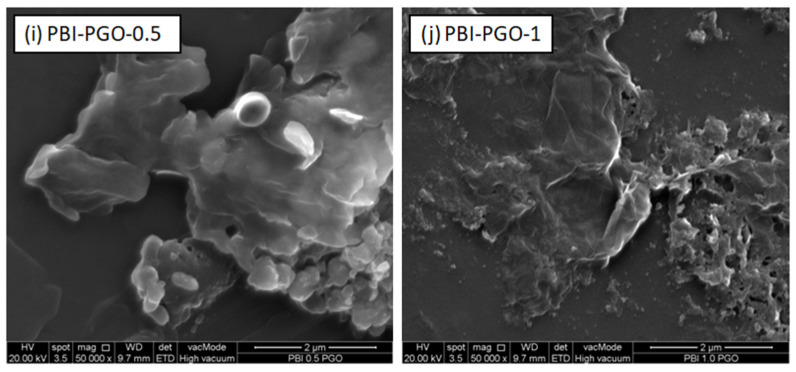
(**a**–**j**) Polybenzimidazole (PBI) and PBI composites with GO, PGO, and SGO at different loading levels.

The surface morphology of the PBI and composite membranes are shown from Figure 4a–j. The pure PBI (Figure 4a) has an overall smooth surface without any cracks and pinholes, consistent with those PBI membranes determined by Uregen et al. [8] and Kuo et al. [38]. Membranes with GO, SGO, and PGO displayed wrinkles and stacked layers over the membrane surface. The surface of the membranes becomes more wrinkled with more agglomerations of the fillers when the filler concentration increases to 0.5 and 1 wt.%, which is caused by the van der Waals forces between the sheet fillers [39,40]. Hydrogen bonding interactions between the fillers and membrane may also be responsible for the wrinkles and stacking on the composite membranes’ surface [41,42].

### 3.3. XRD

**Figure 5:**
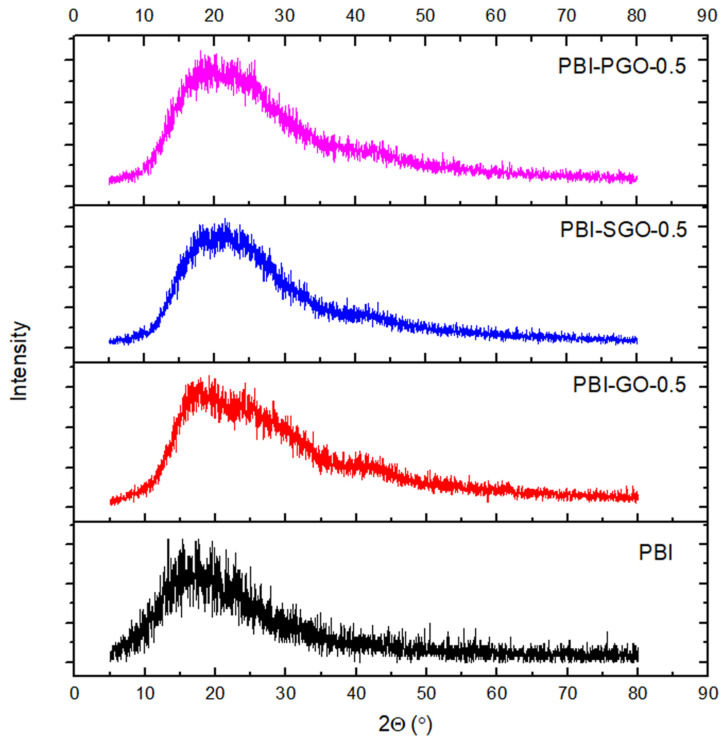
X-ray diffraction (XRD) spectrum of PBI composite membranes.

Figure 5 is the spectrum of the PBI composite membranes with 0.5 wt.% of fillers. Pure PBI has a broad, medium band in the range of 2ϴ = 14 to 25° with the absence of any sharp peaks, indicating that the membrane is mainly amorphous [43]. This band also implies that the PBI polymer chains were arranged in a disordered manner, and there was spacing between the polymer chains to accommodate the phosphoric acid molecules [44]. No significant changes or shifts in the spectrum and no sharp peaks are observed when 0.5 wt.% of each type of fillers was added. Furthermore, the broadband within 2ϴ = 14 to 25° is retained; therefore, the PBI composites maintained their amorphous properties without significant disorientation or reorientation of the PBI’s molecular arrangement.

### 3.4. TGA

**Figure 6:**
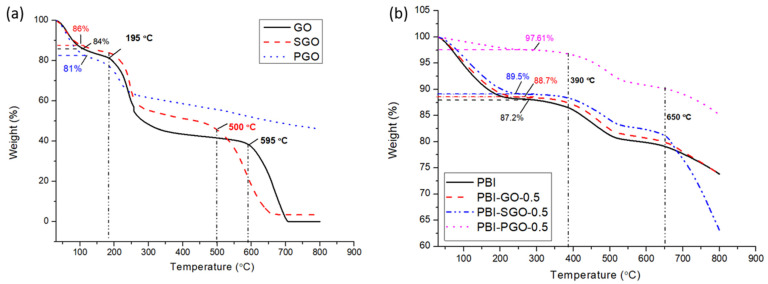
Thermogram of (**a**) fillers, and (**b**) composite membranes.

The thermograms of the fillers and composite membranes under the N_2_ environment are shown in Figure 6a,b, respectively. According to Figure 5a, GO and SGO displayed three-stage weight losses, while there are two stages for PGO. Weight loss up to around 190 °C relates to the loss of absorbed moisture [45]. In the second stage, oxygen-containing functional groups within the three fillers began to decompose at around 195 °C, up until 300 °C. GO shows the highest weight loss, followed by SGO and PGO. The larger residual weight of SGO compared to GO is likely due to the presence of sulfonic acid groups that decompose at a higher temperature. Decomposition of sulfonic acid may begin at around 300 °C, and the breaking of most of the sulfonic acid alongside the C–C and C=C bonds on the graphene plane may have caused the rapid weight loss after 500 °C [24,46]. Meanwhile, the degradation of the graphene plane on GO occurred at 595 °C [23,24,47]. On the other hand, PGO maintained a larger residual weight than GO and SGO after 250 °C, and also does not undergo any further degradation after 500 °C. As indicated in the FTIR spectra (Figure 2a), PGO lacks certain oxygen-containing functional groups, particularly carboxylic acid groups; this may explain its limited weight loss in the second stage. Zelechowska et al. [48], on their thermogram result of PGO, suggested that the replacement of carboxylic acids by phosphonated groups and removal of other oxygen-containing functional groups helps to enhance the PGO’s thermal stability. The absence of further decomposition in PGO may also be due to the phosphonic acid groups undergoing a reversible self-condensation reaction and formation of anhydride P–O–P bonds [49]. These bonds may hold the graphene planes together, thus slowing the decomposition rate of PGO.

The thermogram of PBI and PBI composites without acid-doping is shown in Figure 6b. All membranes displayed a three-stage decomposition pattern. The first stage weight loss is associated with the loss of absorbed moisture and residual solvents [25,31,35,50]. The slight decomposition after 380 °C may be caused by the breaking of N–H and –N= functional groups in the imide rings of PBI [38]. The third stage decomposition which occurred at approximately 650 °C is related to the decomposition of the PBI backbone [51]. The membrane’s residual weight slightly increases upon the addition of GO and SGO, while the presence of PGO appeared to maintain the residual weight of the PBI above 90%. GO and SGO disintegrate after 500 °C and thus its presence in the PBI membrane can sustain the weight of the membrane above 80% before the final degradation after 500 °C. PBI with SGO degrades more rapidly in the third stage, likely due to the faster degradation of SGO. PBI with PGO does not degrade as rapidly compared to those with SGO and GO. A possible cause is the more stable PGO that does not decompose abruptly after 500 °C, thus remaining in the PBI membrane. Additionally, the strong hydrogen bonding that formed between the phosphonic acid groups in PGO with the PBI could also contribute to its thermal strength [49,52]. Overall, the presence of GO, SGO, and PGO can maintain the thermal stability of the membrane to some extent, considering the increase in their residual weights, with the PGO-containing membrane sustaining more than 90% of its weight. Within the operating temperature around 110 to 180 °C, the remaining weight of the membranes is above 80% and is mostly due to the loss of absorbed water and residual solvents, thus these membranes can be considered thermally stable to be utilized as PEM in HTPEMC.

### 3.5. ADL and Dimensional Swelling

**Figure 7:**
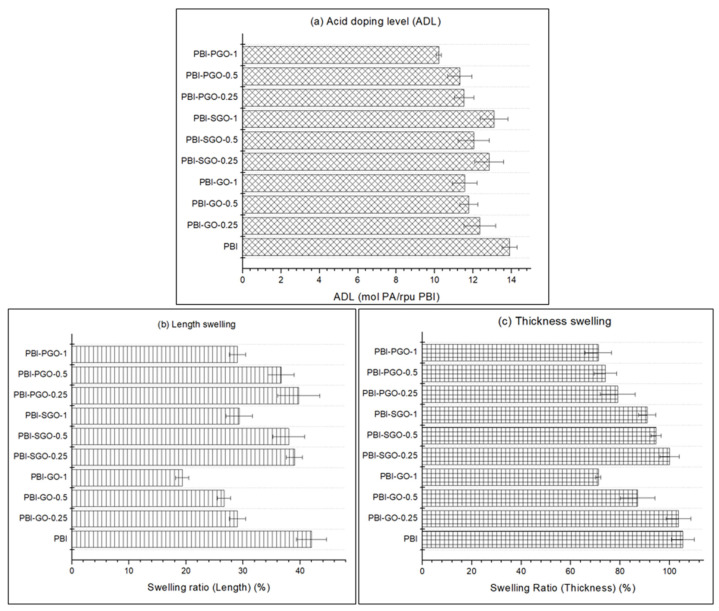
(**a**) Acid doping level (ADL), (**b**) length swelling, and (**c**) thickness swelling of PBI membranes.

The ADL of the PBI membrane plays an important role in its proton conductivity under high temperature and anhydrous conditions. The ADL of PBI and its composites are displayed in Figure 7a. Pure PBI achieved an ADL of around 13.9 mol PA/rpu PBI after doping for four days at room temperature. Introduction of GO, SGO, or PGO into the membrane appeared to lower the ADL. The PBI with GO and PGO was lowered by 2.34 and 3.68 mol PA/rpu PBI, respectively, when 1 wt.% of each filler were present. Interestingly, PBI with SGO displayed a decrease in ADL at first, before increasing again when the concentration of SGO is 1 wt.%. The lower ADL of the PBI composites compared to pure PBI may be the result of the N-H functional groups in PBI interacting with the oxygen-containing functional groups in GO, as well as with the additional sulfonic acid present in SGO and phosphonic groups in PGO. This resulted in lower basicity of PBI where the N–H has been occupied, which prevents binding to more phosphoric acid molecules. Furthermore, the sheet structures of the GO, SGO, and PGO that are lodged in the free volumes of the membrane also limit the available spaces to be occupied by free acids [8,53,54]. On the other hand, a higher concentration of SGO in PBI also increases the amount of hydrophilic ^−^SO_3_H groups that easily form hydrogen bonding with water as well as phosphoric acid molecules, which may cause the ADL to increase slightly [18]. As for the PGO-containing membrane, the results are opposite to that of the Py-PBI/PGO by Abouzari-Lotf et al. [11], since they observed an increase in ADL. There are lesser oxygen-containing functional groups in PGO, and the phosphonic acids are also less sensitive to moisture than sulfonic acid due to their lower acidity [55]. Therefore, it may likely limit the hydrogen bonding that can be formed to retain additional PA molecules or water.

The dimensional swelling in terms of length and thickness direction is shown in Figure 7b,c. PA molecules occupying the free volumes between the polymer layers cause polymer chain separation and membrane expansion, which is usually larger in the thickness direction [6,56,57]. All the composite membranes with GO, SGO, and PGO have smaller dimensional swelling than pure PBI. The swelling restriction can be attributed to the smaller ADL. Additionally, intermolecular interactions formed between the functional groups on the fillers with the N–H, as well as ^−^N= functional groups in PBI, limiting the polymer chain expansion. PBI with 1 wt.% GO showed the least swelling in both length and thickness, likely due to the strong binding provided by the large amounts of GO in the PBI matrix. PBI-SGO membranes swelled more in thickness, which may be caused by their higher ADL than PBI-GO and PBI-PGO. Meanwhile, PBI-PGO has moderate thickness swelling with larger length expansion.

### 3.6. Proton Conductivity

**Figure 8:**
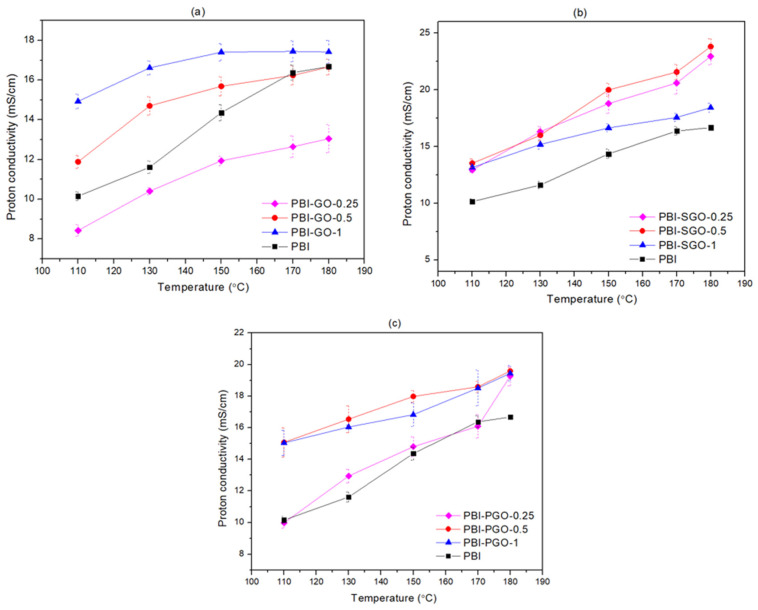
Proton conductivity under anhydrous conditions for (**a**) PBI-GO, (**b**) PBI-SGO, and (**c**) PBI-PGO.

The proton conductivity of all the doped PBI membranes was analyzed through EIS from 110 to 180 °C without humidity, mimicking the HTPEMFC environment. Figure 8a–c show the proton conductivity of the PBI with GO, SGO, and PGO, respectively. All membranes displayed a steady increase in proton conductivity with increasing temperature, as increasing temperature results in faster kinetics and movement of protons through the membrane. The conductivity of pure PBI is 16.7 mS/cm at 180 °C. The composite membranes have larger conductivity than pure PBI, except PBI with 0.25 wt.% of GO, throughout the whole temperature range. In Figure 8a, the conductivity of PBI-GO increases with the higher loading of GO. At 180 °C, the conductivity of PBI-GO-1 is the highest at 17.4 mS/cm, which is about 4.2% higher than pure PBI, and was achieved despite the smaller ADL of the membrane. This can be attributed to the oxygen-containing functional groups such as hydroxyl, epoxy oxygen, and carboxylic acid on GO participating as additional proton transfer sites by forming a hydrogen-bonding network with the N–H groups in PBI and with PA molecules, hence creating new proton transfer pathways [58]. Uregen et al. [8] have revealed the increase in proton conductivity of PBI with GO, even though there were no significant changes to the membranes’ ADL, where the oxygen-containing functional groups contribute as additional proton transfer sites. The smaller proton conductivity of PBI-GO-0.25 may be due to the sheet structure of GO making the proton transfer pathway more tortuous, slowing down its diffusion [41]. The very small amount of GO may not provide sufficient additional proton transfer sites.

PBI-SGO membranes have higher proton conductivity than pure PBI, as shown in Figure 8b. The conductivity increases with increasing SGO loading from 0.25 to 0.5 wt.%, before dropping at 1 wt.% loading. Proton conductivity of PBI-SGO-0.25 and PBI-SGO-0.5 are also near comparable to each other, with the former exceeding the latter after 150 °C. PBI-SGO-0.5 achieved the highest proton conductivity (23.8 mS/cm) among all composites at 180 °C, which is approximately 42.5% higher than pure PBI. Sulfonic acid (^−^SO_3_H) present alongside oxygen-containing functional groups also form new proton transfer pathways with the N-H of PBI through the hydrogen bonding network. Hydrophilic ^−^SO_3_H groups tend to absorb surrounding moisture that can potentially boost the proton conductivity under low humidity or anhydrous conditions, even with the PBI-SGO’s smaller ADL than pure PBI [9,10,19,59]. ^−^SO_3_H is also highly acidic, which can readily bind to protons. Additionally, the PBI-SGO-0.5′ ADL is larger than those with GO or PGO, therefore containing more medium for proton transfer. Yusoff et al. [10] have also reported the increase in PBI proton conductivity by the addition of SGO, attributed to the ^−^SO_3_H acting as proton transfer sites and the increase in ADL. The drop in conductivity in PBI-SGO-1 may be due to the aggregation of SGO and the tortuous proton conductive pathways caused by the sheet structures of the fillers.

PBI with 0.25 wt.% PGO has comparable proton conductivity to pure PBI, as seen in Figure 8c. The PBI-PGO membrane shows a similar trend to that of PBI-SGO, where the conductivity increases up to 0.5 wt.% PGO loading, before dropping when the loading is 1 wt.%. At 180 °C, the conductivity of PBI-PGO at all PGO loading levels is very close, with PBI-PGO-0.5 achieving the highest conductivity of 19.6 mS/cm, about 17.4% higher than the conductivity of pure PBI. Similar to the effects of GO and SGO, the additional phosphonic acid groups present alongside oxygen-containing functional groups contribute to the formation of new proton transfer pathways through hydrogen bonding networks [11]. Furthermore, phosphonic acid groups on PGO also have the same acidity as the phosphoric acid embedded in the membrane (pKa = 2–3) [55]. Blending between phosphonic groups with the phosphoric acid molecules and phosphate ions may result in more uniform proton-conducting channels through the membrane. This appears beneficial in improving the PBI’s proton conductivity, despite the PBI-PGO membranes also having the lowest ADLs among composite membranes. Abouzari-Lotf et al. [11] have shown that PGO functions to improve the proton conductivity of the pyridine-functionalized PBI membrane; however, with a slight increase in ADL. This work shows a contrast in ADL as it was reduced. However, adding PGO was shown to increase the conductivity of PBI. The work of Zhang et al. [13] on PGO in Nafion membrane demonstrated that the presence of PGO enhances the proton conductivity of Nafion even at a low relative humidity of 40% and at high temperature up to 110 °C. This work further highlights the advantage of phosphonic acid-functionalized GO in providing effective proton transfer pathways, especially for low humidity and high-temperature conditions.

**Figure 9:**
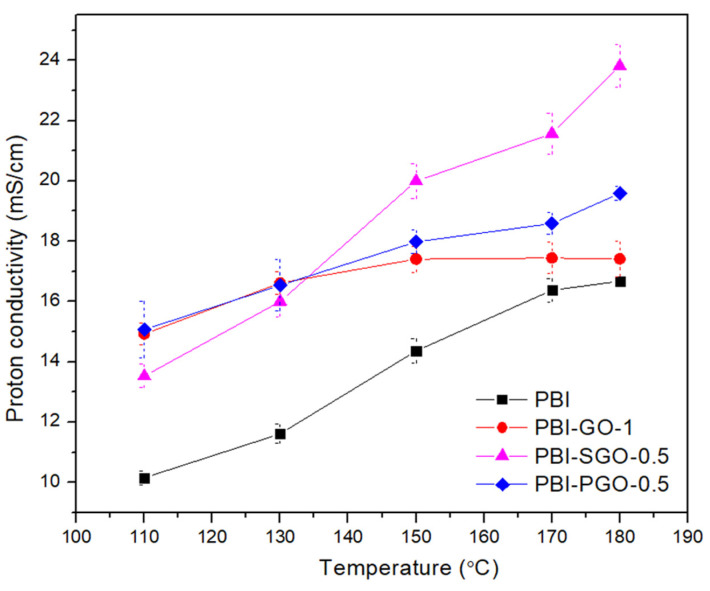
Proton conductivity of the selected composites compared with pure PBI.

To study the differences in the influence of GO, SGO, and PGO towards the proton conductivity of PBI, the composites showing the highest conductivity are selected from each type of filler, as shown in Figure 9. All selected composites have higher proton conductivity than pure PBI throughout the whole temperature range. At 110 °C, the conductivity of PBI-GO-1 and PBI-PGO-0.5 are similar to each other and are both larger than PBI-SGO-0.5. The latter membrane overtakes the conductivity of the first two composites after 150 °C. PBI-SGO-0.5 has the highest conductivity at 180 °C (23.8 mS/cm), followed by PBI-PGO-0.5 (19.6 mS/cm) and PBI-GO-1(17.4 mS/cm). Therefore, the proton conductivity of PBI with sulfonic acid and phosphonic acid-functionalized GO is better than that of PBI with normal GO. The high proton conductivity of PBI with SGO can be attributed to the membrane’s high ADL, the hydrophilic properties of SGO, and the high acidity of the sulfonic acid group (−SO_3_H), with a pKa < 1, which means these groups release and receive protons more readily than carboxylic (pKa = 3–4), and phosphonic groups (pKa = 2–3) [55]. Yet, it should be noted that at 110 °C, the conductivity improvement is smaller for PBI-SGO-0.5 (~32%) compared to PBI-GO-1 (~46%) and the following PBI-PGO-0.5 (~48%), indicating that the PBI with SGO would require higher activation energy. While PBI-PGO-0.5 has smaller conductivity than PBI-SGO-0.5 at 180 °C, it is still higher than PBI-GO-1, despite having the least ADL among selected membranes. The good proton conductivity of PBI-PGO-0.5 is attributed to the uniform acidity, as well as the phosphonic acid’s stronger acidity than carboxylic acids and hydroxyl groups.

**Figure 10:**
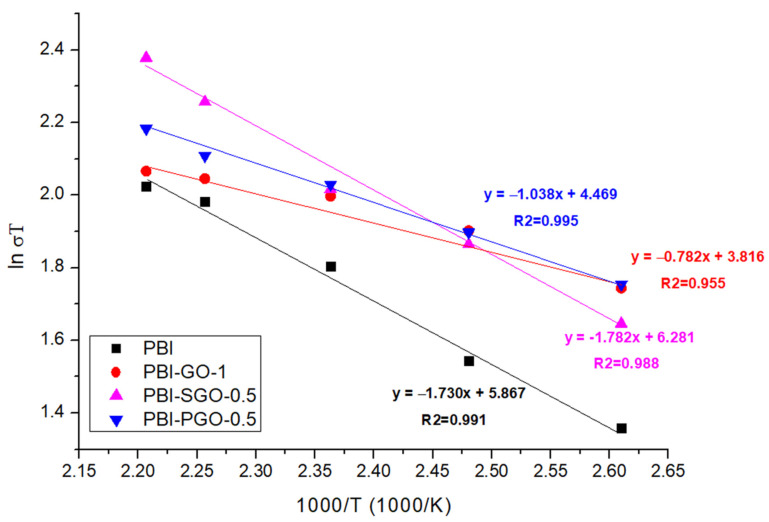
Arrhenius plot of selected PBI membranes.

The proton conductivity of the membrane can be correlated with its activation energy through the Arrhenius equation. The activation energy (*E_a_*) for proton conduction is the minimum energy required for the protons to jump from one ionic domain to another [8,60]. The *E_a_* was determined from the slope of the Arrhenius plot obtained based on the linear equation below [10,37]:

(6) $$\ln\left(\sigma T\right)=\ln\sigma_{o}-\left(\frac{E_{a}}{R}\right)\left(\frac{1000}{T}\right)$$

where *σ* is the proton conductivity (S/cm), *σ_o_* is the pre-exponential factor (S/K cm), *R* is the ideal gas constant (8.314 J/mol.K), *T* is the temperature in Kelvin (K) and *E_a_* is the activation energy (kJ/mol). The estimated *E_a_* was calculated from the slope of the Arrhenius plot shown in Figure 10, while the value of *E_a_* is tabulated in Table 1.

**Figure 11:**
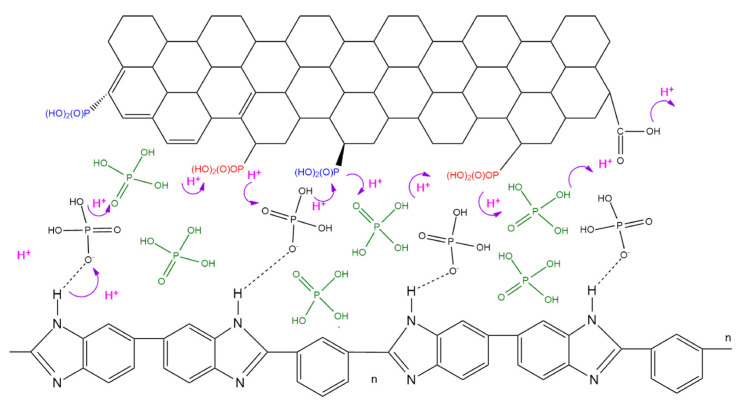
Schematic diagram on the proposed Grotthus mechanism of proton transfer between PA-doped PBI and PGO.

In Figure 10, the steep slope of the PBI-SGO-0.5 membrane corresponds to its larger *E_a_* value compared to PBI-GO-1 and PBI-PGO-0.5, plus it is also slightly higher than that of pure PBI, implying that more energy is required to achieve a faster proton transfer rate. *E_a_* of PBI-GO-1 is the smallest, followed by PBI-PGO-0.5. Under anhydrous conditions, proton transfer in PA-doped PBI generally follows the Grotthuss (proton-hopping) mechanism, where the protons jumped between the ionized N-H site, PA molecules, and H_2_PO_4_^+^/H_2_PO_4_^−^ ion pairs. The data for all membranes fits well in the Arrhenius plot with little irregularities, suggesting that the dominant proton-conducting mechanism is the Grotthuss mechanism [61]. Activation energy greater than 14 to 40 kJ/mol is also attributed to the Grotthuss mechanism [37,62]. Figure 11 illustrates this proposed proton transfer between PA-doped PBI and PGO. *E_a_* value smaller than 10 kJ/mol suggests a rapid proton transfer occurs in the membrane due to additional continuous proton transfer channels. Furthermore, the secondary vehicular mechanism may also present alongside the Grotthuss mechanism, where there is a self-diffusion of protons that are bound to PA or any available water molecules throughout the membrane [8,59,63,64,65].

The varying effects that the GO, SGO, and PGO have towards *E_a_* can be attributed to the individual aspects of the fillers, the interactions between fillers and the PBI chain, and their functional groups’ ionization ability. New proton transfer channels were created between the oxygen-containing functional groups and additional phosphonic acid groups, facilitating the transfer of protons in PBI-GO-1 and PBI-PGO-0.5. A large concentration of oxygen-containing functional groups in GO connect with the N-H and PA molecules that shortened the distance for proton hopping, forming narrow but continuous transfer channels, hence minimizing the energy required for protons to switch from one ionic domain to another [66,67]; conversely, for the PBI with PGO, the uniform acidity along the membrane helps ease the proton transfer. Higher *E_a_* of PBI-PGO-0.5 than PBI-GO-1 may be due to the lesser oxygen-containing functional groups in PGO that can form a more interconnected hydrogen bonding network. Nevertheless, phosphonic acid from PGO can effectively facilitate proton transfer that leads to higher conductivity of the PBI with PGO than with GO. Phosphonic acid and phosphate ions can function as better proton conductors than sulfonic acid and carboxylic acids under anhydrous conditions, due to the acids’ ability to form stronger hydrogen bonding, have amphoteric properties, and also retain moisture, even when their acidity is smaller than that of sulfonic acid [11,13,49,52]. Their good ionization abilities help facilitate proton transfer when there is a lack of moisture, therefore lowering the *E_a_*. In contrast, the high *E_a_* of PBI-SGO-0.5 may be likely due to the poorer ionization capabilities of −SO_3_H groups in SGO under anhydrous conditions [15,68]. While the membrane may have attracted a significant amount of water initially, water is gradually removed above 100 °C. Studies on Nafion membranes [13,68,69] and H_2_SO_4_-doped PBI membranes [14] have shown that the proton conductive abilities of ^−^SO_3_H are highly dependent on humidity, which significantly increases when the relative humidity levels exceed 50%. The proton conductivity of PBI-SGO-0.5 surpasses the conductivity of PBI-GO-1 and PBI-PGO-0.5 after 150 °C, as seen from Figure 9, implying that ^−^SO_3_H ionizes more slowly under dry conditions, but may become thermally activated at certain temperatures and start to ionize at a faster rate when more energy is gained. Requiring low *E_a_* to achieve high proton conductivity could help in heat energy utilization in HTPEMFC. Furthermore, reaching higher proton conductivity at the lower temperature between 150 to 170 °C may also benefit in terms of a faster startup of the cell and limiting the acid loss due to PA evaporation. Considering high conductivity with low *E_a_*, the PBI-PGO-0.5 appears to hold this advantage.

### 3.7. Acid Retention

**Figure 12:**
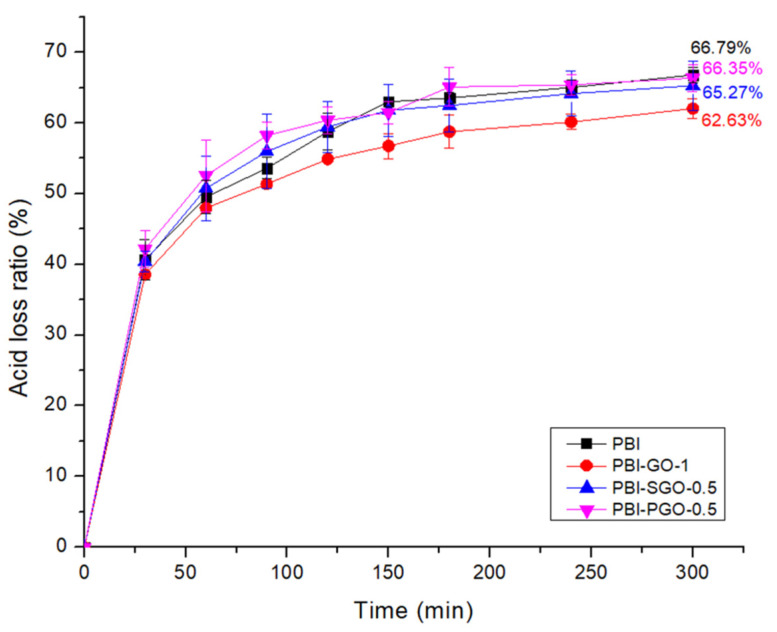
Phosphoric acid loss ratio of selected PBI composites.

Good acid retention in PBI is important in ensuring the long-term proton conductivity of the membrane. The phosphoric acid loss of selected PBI composites with the highest proton conductivity is as shown in Figure 12. All membranes rapidly lost around 38 to 42% of the acid in the first 30 min before gradually stabilizing. SGO and PGO at 0.5 wt.% in PBI did not show any large changes to acid retention of pure PBI. Addition of 1 wt.% GO was able to lower the acid loss by about 4.16%, which can be attributed to the large quantities of GO sheets occupying the PBI matrix, making the internal structures denser and slowing the diffusion rate of acid out of the membrane. Furthermore, the high concentration of oxygen-containing functional groups in GO helps bind the PA molecules through hydrogen bonding, which also slows down the leaching [8,19].

## 4. Conclusions

The introduction of GO, SGO, and PGO into PA-doped PBI membranes has been shown to enhance certain properties, namely thermal stability and proton conductivity. Reduction in ADL by around 2 to 4 mol PA/rpu PBI was observed in the filler-containing membranes. This also lowers the dimensional swelling of the membranes. Thermal analysis on the overall thermal stability shows that the PGO-containing PBI was able to retain more than 90% of their weight within the HTPEMFC operating temperature. The key property, which was the proton conductivity, was also improved despite the suppression of the composite membrane’s ADL. PA-doped PBI with 0.5 wt.% SGO acquired the highest proton conductivity at 180 °C, under anhydrous conditions, at 23.8 mS/cm. This is followed by the membrane with 0.5 wt.% PGO at 19.6 mS/cm, attributed to the presence of acidic proton-conducting sulfonic acid and phosphonic acid functional groups that functions as additional effective proton transfer groups. While the conductivity of the PBI with SGO is the highest, the PBI with PGO required a lower activation energy and its conductivity was better than that with GO. This was achieved despite the PBI with PGO showing the least ADL among the composites, contributed by the good ionization ability of phosphonic acid under anhydrous conditions and the uniform acidity across the membrane. Considering the significant enhancement to proton conductivity, lower activation energy, and good thermal stability of the PGO-containing PBI, the PGO is considered a potential effective filler for the PBI membrane applied for HTPEMFC.

## Figures and Tables

**Table 1:** The activation energy of the selected PBI membranes.

Membranes	*E_a_* (kJ/mol)
PBI	14.38
PBI-GO-1	6.50
PBI-SGO-0.5	14.82
PBI-PGO-0.5	8.63
